# The role of serum α-Klotho levels in preventing hearing impairment among middle-aged and older adults: insights from a nationally representative sample

**DOI:** 10.3389/fnagi.2024.1415494

**Published:** 2024-11-14

**Authors:** Siyuan Wang, Wen Sun, Chan Ding, Wenxin Zhou, Min Zhang, Huadong Xu

**Affiliations:** ^1^School of Public Health, Zhejiang Provincial People's Hospital (Affiliated People's Hospital), Hangzhou Medical College, Hangzhou, Zhejiang, China; ^2^School of Medical Humanities and Management (School of General Practice Competency Education), Hangzhou Medical College, Hangzhou, Zhejiang, China

**Keywords:** Klotho, adults, hearing thresholds, hearing loss, restricted cubic splines

## Abstract

**Background:**

The Klotho gene is implicated in suppressing aging phenotypes and influencing age-related diseases. Previous studies have delved into its connection with different diseases, yet the association between Klotho and hearing loss has rarely been examined. A recent population study explored the relationship between serum Klotho and hearing loss, but it had certain limitations. This study aims to analyze the link between serum α-Klotho levels and hearing thresholds, as well as the risk of hearing loss.

**Methods:**

A total of 1,762 adults aged 40–69 years were selected from the 2011–2012 National Health and Nutrition Examination Survey (NHANES). Data on audiometry, serum α-Klotho levels, and relevant covariates were gathered. Statistical analyses, including linear and logistic regression, assessed the relationships of serum α-Klotho levels with hearing outcomes.

**Results:**

Increased serum α-Klotho levels were correlated with diminished hearing thresholds and a lower risk of hearing loss. Quartile analysis revealed a significant trend, where elevated α-Klotho levels were linked to better auditory outcomes. Adjusted models controlled for various covariates, affirming the robustness of the findings. Non-linear associations were not observed.

**Conclusion:**

This study provided novel evidence of a negative association between serum α-Klotho and hearing impairment in adults aged 40–69. Our results suggested a protective role of serum α-Klotho on adults with hearing loss.

## 1 Introduction

Klotho is a gene involved in the suppression of multiple aging phenotypes and is known to participate in a signaling pathway that regulates the incidence of aging and age-related diseases (Kuro-o et al., 1997). Modulating Klotho activity has emerged as a promising therapeutic approach for aging-related conditions, including chronic kidney disease (CKD). Consequently, several senotherapeutic approaches have been developed with the aim of directly or indirectly impacting Klotho expression (Buchanan et al., 2020). Aberrant low expression of the Klotho gene has been observed in a range of aging-related diseases (Zhou et al., 2021).

Hearing impairment, the second most prevalent disability in the United States, affects approximately 17% of the population and holds significant implications for the aging demographic (Nieman and Oh, 2020; Zazove et al., 2023). Age-related hearing loss (ARHL) is a progressive condition characterized by bilateral and symmetrical sensorineural hearing loss. It primarily manifests at higher frequencies due to the cumulative impact of age on the auditory system (Agrawal et al., 2008). The onset typically occurs around the age of 50 and involves changes in structure and function in the cochlea, auditory nerve, and central auditory cortex (Deal et al., 2017; Völter et al., 2018). Initially, the higher frequencies are predominantly affected, followed by the midrange and lower frequencies, resulting in elevated hearing thresholds and compromised frequency resolution (Yamasoba et al., 2013). Numerous studies have unequivocally demonstrated the profound impact of hearing loss on the individual quality of life. It diminishes social interactions, contributes to feelings of loneliness and cognitive impairments, and elevates the risk of depression and dementia (Powell et al., 2021; Sun et al., 2021; Zazove et al., 2023). Numerous factors, including vascular occlusion, immune response, and abnormal cellular stress responses in the cochlea as well as viral infections, have been implicated in the etiology of hearing loss (Chen et al., 2019). Furthermore, emerging research has linked hearing loss to reduced kidney function, smoking, diabetes, cardiovascular disease, and other middle-aged individuals' risk factors (Lin et al., 2020; Liu et al., 2020; Baiduc et al., 2023; Deng et al., 2023). Additionally, increases in body mass index (BMI) had exhibited positive associations with the risk of hearing loss (Yang et al., 2020).

Several previous studies have tried to examine the expression of the Klotho protein not only in the stria vascularis but also in the spiral ligament of the inner ear, and its relationship with hearing loss *in vivo* (Kamemori et al., 2002; Takumida et al., 2009; Wang Y. et al., 2023). Mouse experiments have suggested that Klotho may play a significant role in ion homeostasis in the endolymph (Kamemori et al., 2002). However, subsequent research has shown that normalizing 1,25(OH)_2_D_3_ levels could alleviate hearing loss in Klotho-deficient mice, appearing that hearing impairment might be indirectly mediated by high systemic 1,25(OH)_2_D_3_ rather than by a direct lack of Klotho expression (Carpinelli et al., 2011). To date, the relationship by which Klotho deficiency leads to hearing loss remains controversial.

In recent years, numerous studies have utilized NHANES statistical data to delve into the associations between serum α-Klotho and various health factors, such as oral health, blood lead levels, blood cadmium levels, and alcohol consumption, specifically among American adults (Chen et al., 2022; Kim et al., 2022; Jiang et al., 2023). However, despite this wealth of research, there is a notable scarcity of studies examining the relationship between Klotho and hearing threshold, as well as the risk of hearing loss. A recent NHANES study investigated the relationship between serum Klotho and hearing loss using a sample of 1,781 individuals aged 20–69 (Zhou et al., 2023). Our investigation, through a meticulous analysis of NHANES statistics, bridges this research gap by presenting compelling evidence that elevated levels of α-Klotho are linked to lower hearing thresholds and a diminished risk of hearing loss.

## 2 Methods

### 2.1 Study design and population

The NHANES is a nationally representative survey of the non-institutionalized US civilian population conducted by the Centers for Disease Control and Prevention (CDC) (Xu et al., 2021a; Zhong et al., 2023a). The interview process of this comprehensive study covers various aspects including demographics, socioeconomic factors, dietary habits, and health-related questionnaire responses. In addition, NHANES includes the administration of laboratory tests and physiological examinations conducted by expertly trained medical personnel. The datasets generated from the NHANES are publicly accessible through the NHANES website. Ethical approval for the research was obtained from the NCHS Ethics Review Board, and all participants provided written informed consent. The NHANES data is widely used to assess risk factors for various diseases in adults (Xu et al., 2021b; Xu and Bo, 2022; Zhang et al., 2023; Zhong et al., 2023b).

**Figure 1:**
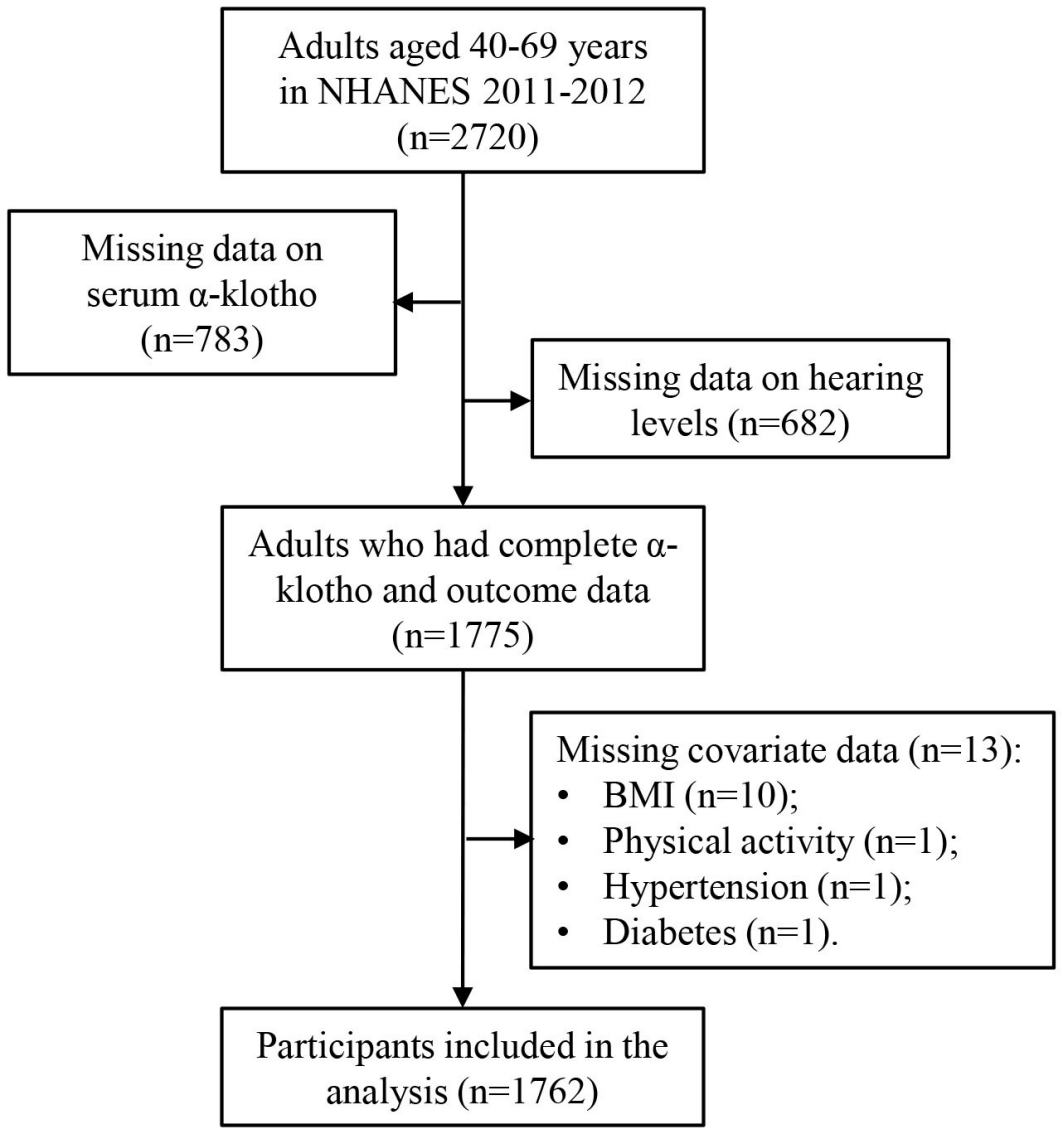
Flow diagram illustrating the selection of the study population.

To optimize the sample size, data for this analysis were extracted from the 2011 to 2012 NHANES dataset. Initially, a total of 2,720 participants aged 40–69 years were enrolled during this period, as audiometry examinations were conducted only in individuals aged 20–69 years, and serum Klotho protein concentrations were measured only in individuals aged 40–79 years. After excluding individuals with missing data on serum α-Klotho concentrations (n = 783) and incomplete information on hearing loss (*n* = 682), the final analysis comprised 1,775 participants with complete α-Klotho and outcome data. Furthermore, participants with missing covariate information, such as BMI (*n* = 10), physical activity (*n* = 1), hypertension (*n* = 1), and diabetes (*n* = 1), were removed, resulting in a final sample size of 1,762 participants. The participant selection process is shown in Figure 1.

### 2.2 Audiometric measurement

Audiometric assessments were conducted as part of the NHANES project, following a standardized protocol provided by the NCHS. The thresholds, measured in dB, represent the quietest sound that can be heard at each frequency, and the Pure Tone Average (PTA) is calculated by the average of hearing thresholds at these frequencies. In the NHANES project, PTA hearing thresholds were measured by trained examiners for both ears at seven frequencies (500, 1,000, 2,000, 3,000, 4,000, 6,000, and 8,000 Hz). Additionally, a frequency of 1 kHz was assessed twice for each ear, and if a disparity exceeding 10 dB was observed between the results, the participant was excluded, and their initial responses were not analyzed (Liu et al., 2023).

Previous research has distinguished two categories of hearing: speech-frequency hearing threshold (SFHT) and high-frequency hearing threshold (HFHT). For SFHT, the average frequencies at 0.5, 1, 2, and 4 kHz were calculated, while for HFHT, the average frequencies at 3, 4, and 6 kHz were determined (Li et al., 2022). In this study, an outcome variable was defined as a PTA exceeding 25 dB in either ear, indicating a significant hearing loss according to the World Health Organization Grades of Hearing Impairment (Bainbridge et al., 2008; Olusanya et al., 2019).

### 2.3 Serum Klotho measurements

Serum samples were obtained at the mobile examination center and subsequently stored at −80°C until analysis. The measurement of Klotho was conducted using an enzyme-linked immunosorbent assay (ELISA) kit. Each sample underwent duplicate analysis, and the average of the two concentrations was considered the final value (Zhang et al., 2022). For quality control, two samples with varying Klotho concentrations were included in each ELISA plate. Detailed information regarding the laboratory methodology and quality assurance can be accessed at https://wwwn.cdc.gov/Nchs/Nhanes/2011-2012/SSKL_G.htm.

### 2.4 Covariates

Covariates including age, sex, race/ethnicity, marital status, BMI, physical activity, diabetes mellitus, serum cotinine, hypertension, and noise exposure condition were selected as covariates according to previous literatures (Xu et al., 2020; Guan G. et al., 2023; Guan Z. et al., 2023). Exposure to loud noise/music was collected based on questionnaires. Serum cotinine level was selected as a biomarker for tobacco exposure.

### 2.5 Statistical analysis

All statistical analyses were performed using R language (version 4.2.1, R Foundation for Statistical Computing), and “survey” and “RMS” packages were used in the R platform. Statistical methods such as *t-*tests or analysis of variance (ANOVA) were applied to compare the differences in serum α-Klotho levels between or among different groups. The distributions of hearing thresholds as well as hearing loss across quartiles of serum α-Klotho levels were analyzed using ANOVA for trend analysis. Serum α-Klotho levels were natural logarithm (ln)-transformed to normalize distributions, and regression models were used to model these outcomes. Multiple linear regression models assessed the relationships of serum α-Klotho levels with SFHT and HFHT. Serum α-Klotho levels were also modeled into quartiles to better capture non-linear relationships. The relationships of serum α-Klotho with speech-frequency hearing loss (SFHL) and high-frequency hearing loss (HFHL) across quartiles of serum α-Klotho levels were then analyzed by comparing them to the lowest quartile. In these analyses, *p*-values for a linear trend were calculated by fitting the α-Klotho quintile as an ordinal categorical variable. For SFHL and HFHL, multiple logistic regression models were used to assess the relationships of serum α-Klotho with SFHL and HFHL. Finally, restricted cubic splines (RCS) were applied to simulate the dose-response relationships and potential nonlinear associations. All models were adjusted for age, sex, race/ethnicity, BMI, physical activity, marital status, serum nicotine, hypertension, diabetes, and exposure to loud noise/music. Statistical significance was defined as *p* < 0.05.

## 3 Results

### 3.1 Characteristics of the study participants

The baseline characteristics of a total of 1,762 participants in the study is listed in Table 1. Among the participants, 49.3% were male with a mean α-Klotho concentration of 878.32 ± 306.42 pg/mL, and 50.7% were female with a mean α-Klotho concentration of 926.20 ± 340.16 pg/mL (*p* = 0.002), indicating that females had significantly higher serum α-Klotho levels. There was a statistically significant difference in α-Klotho levels across different racial/ethnic groups (*p* = 0.038). No significant differences were observed in α-Klotho levels across different BMI, physical activity, marital status, serum cotinine, diabetes, hypertension, or noise exposure groups.

**Table 1:** Serum α-Klotho levels according to the characteristics of the study participants.

**Characteristic**	**n**	**(%)**	**Serum α-Klotho levels (pg/mL)**	***p*-value**
**Age, years**				0.787
40–49	570	32.3	910.12 ± 327.76	
50–59	616	35.0	897.52 ± 316.92	
60–69	576	32.7	900.55 ± 329.66	
**Sex, %**				0.002
Male	869	49.3	878.32 ± 306.42	
Female	893	50.7	926.20 ± 340.16	
**Race/ethnicity, %**				0.038
Mexican American	182	10.3	884.19 ± 302.12	
Non-Hispanic White	640	36.3	881.39 ± 310.68	
Non-Hispanic Black	490	37.8	935.75 ± 375.28	
Others	450	25.5	904.96 ± 290.02	
**Marital status, %**				0.191
Married/cohabiting	1,095	62.1	896.65 ± 317.05	
Widowed/divorced/separated	471	26.7	899.91 ± 336.45	
Never married	196	11.1	942.18 ± 337.40	
**BMI, kg/m** ^2^				0.213
< 25	430	24.4	925.42 ± 358.43	
25–29.9	611	34.7	900.36 ± 325.55	
≥30	721	40.9	890.86 ± 301.92	
**Moderate physical activity, %**				0.129
No	1,192	67.7	910.58 ± 326.65	
Yes	570	32.3	885.44 ± 320.51	
**Diabetes, %**				0.679
No	1,482	15.9	903.98 ± 325.93	
Yes	280	84.1	895.23 ± 318.92	
**Hypertension, %**				0.063
No	1,001	56.8	915.11 ± 327.64	
Yes	761	43.2	886.10 ± 320.28	
**Serum cotinine, %**				0.788
< LOD	472	26.8	906.02 ± 329.30	
≥LOD	1,290	73.2	901.33 ± 329.30	
**Exposure to loud noise/music, %**				0.422
No	1,625	92.2	900.78 ± 327.20	
Yes	137	7.8	923.99 ± 294.47	

### 3.2 Distributions of hearing profiles according to the serum Klotho levels

Table 2 depicts the distributions of hearing thresholds (SFHT and HFHT) and hearing loss (SFHL and HFHL) across quartiles of serum Klotho levels. The mean ± standard deviation (SD) values were used to present hearing thresholds for different groups. We observed significant differences in hearing thresholds among different α-Klotho quartile groups. For SFHT, the hearing threshold showed a downward trend as serum α-Klotho levels increased, with a *p*-for-trend value of 0.006 across various groups. Similarly, we observed a decreased trend in the HFHT, with a *p*-for-trend value of 0.003 across various groups, indicating that higher serum α-Klotho levels were associated with lower hearing thresholds. For SFHL, the number of adults with SFHL decreased as serum α-Klotho levels increased, and the *p*-for-trend value was 0.007. Similarly, we observed a decreased trend in the number of adults with HFHL, with a *p*-for-trend value of 0.011, indicating that higher serum α-Klotho levels were associated with lower hearing loss.

**Table 2:** The distributions of hearing thresholds and hearing loss by quartiles of serum α-Klotho levels.

**Variables**	**Total**	**Serum** α**-Klotho (pg/mL)**				***p* for trend**
		**Q1 (**<**688.8)**	**Q2 (688.8–848.8)**	**Q3 (849.1–1,056.3)**	**Q4 (**>**1,056.3)**	
**Hearing thresholds**						
SFHT (dB)	16.73 ± 11.01	18.11 ± 11.31	16.92 ± 11.52	16.32 ± 10.10	15.57 ± 10.93	0.006
HFHT (dB)	26.14 ± 16.63	28.26 ± 17.40	26.71 ± 16.60	25.52 ± 16.10	24.07 ± 16.15	0.003
**Hearing loss**						
SFHL, *n* (%)	389 (100%)	119 (30.6%)	104 (26.7%)	84 (21.6%)	82 (21.1%)	0.007
HFHL, *n* (%)	829 (100%)	226 (27.3%)	222 (26.8%)	198 (23.9%)	183 (22.1%)	0.011

### 3.3 Associations of serum Klotho with hearing thresholds

**Figure 2:**
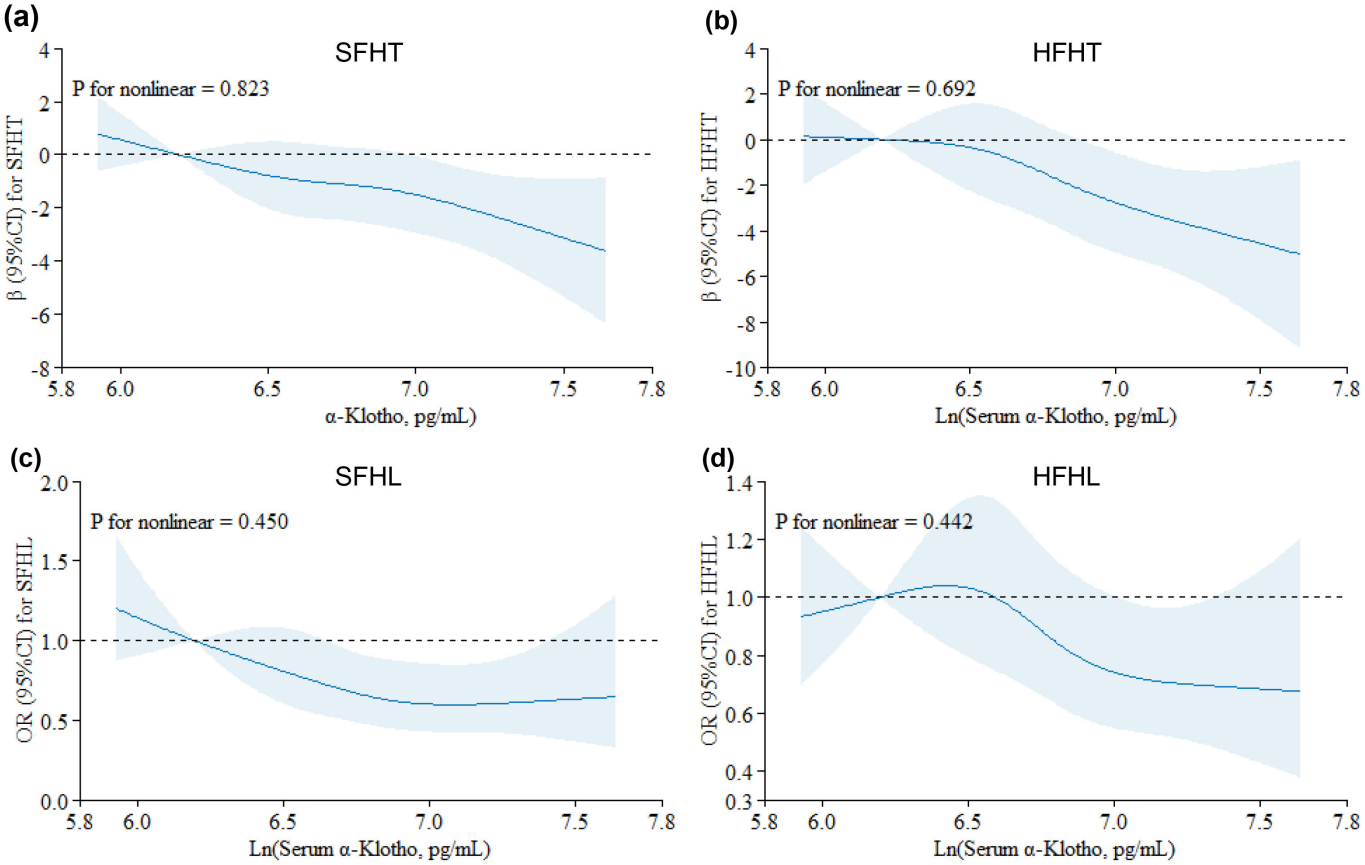
Restricted cubic spline (RCS) plots for the associations of serum α-Klotho levels with hearing thresholds and hearing loss. Graphs show βs for SFHT **(a)** and HFHT **(b)** and ORs for SFHL **(c)** and HFHL **(d)** according to In-transformed serum α-Klotho levels adjusted for age, sex, race/ethnicity, BMI, physical activity, marital status, serum cotinine, hypertension, diabetes, and exposure to loud noise/music. Solid lines indicate βs or ORs, and shadow shape indicates 95% CIs.

Table 3 presents the associations of serum α-Klotho levels with hearing thresholds. The results indicated that every one-unit increase in the ln-transformed serum α-Klotho levels induced a decrease of SFHT by 2.05 dB (*p* = 0.004). Similarly, for every one-unit increase in the natural logarithm of serum α-Klotho levels, HFHT decreased by 2.48 dB (*p* = 0.016). Serum α-Klotho levels were then divided into four quartile groups. Compared to the reference group (Q1, the lowest α-Klotho quartile), both SFHT and HFHT showed decreases in the upper quartiles. This indicated that higher α-Klotho levels were associated with greater reductions in hearing thresholds. RCS curves were generated to examine the dose-response relationship of α-Klotho levels with hearing thresholds (Figures 2a, b). The curves showed a trend of decreasing hearing thresholds with increasing α-Klotho levels, but no non-linear relationship was observed.

**Table 3:** Association of serum α-Klotho levels with hearing thresholds in the study population.

**Serum α-Klotho (pg/mL)**	**n**	***β*** **(95%CI)**, ***p-*****value**	
		**SFHT (dB)**	**HFHT (dB)**
Continuous ln-transformed	1,762	−2.05 (−3.46, −0.64), 0.004	−2.48 (−4.49, −0.47), 0.016
**Quartiles**			
Q1 (< 688.8)	442	Reference	Reference
Q2 (688.8–848.8)	439	−1.34 (−2.68, −0.06), 0.047	−2.15 (−4.05, −0.24), 0.027
Q3 (849.1–1056.3)	441	−1.59 (−2.93, −0.25), 0.020	−2.47 (−4.37, −0.56), 0.018
Q4 (>1056.3)	440	−2.02 (−3.36, −0.68), 0.003	−2.75 (−4.66, −0.85), 0.005
*p* for trend		0.003	0.005

Multiple linear regression models were adjusted for age, sex, race/ethnicity, BMI, physical activity, marital status, serum cotinine, hypertension, diabetes, and exposure to loud noise/music.

### 3.4 Hearing loss risk assessment

Table 4 presents adjusted odds ratios (*OR*s) and their 95% confidence intervals (*CI*s) for the relationship between serum α-Klotho levels and hearing loss in the study population. For SFHL, the OR for continuous ln-transformed α-Klotho levels was 0.62 (95% CI: 0.43, 0.89; *p* = 0.010); for HFHL, the OR was 0.76 (95% CI: 0.55, 1.04; *p* = 0.085. OR values < 1 suggested that each unit increase in In-transformed α-Klotho levels was associated with a reduced risk of hearing loss. Serum α-Klotho levels were then divided into quartiles, with Q1 (< 688.8 pg/mL) serving as the reference group. The trend *p*-values for SFHL and HFHL assessed whether there was a linear trend in hearing loss across the quartiles of α-Klotho levels. Quartile analysis further supported the relationship between serum α-Klotho levels and hearing loss, with higher quartiles of α-Klotho levels having lower odds of hearing loss. These findings suggested that α-Klotho levels might play a protective role in hearing health. RCS curves were generated to assess the dose-response relationships between serum α-Klotho levels and hearing loss. However, no non-linear associations between α-Klotho levels and hearing thresholds were observed (Figures 2c, d).

**Table 4:** Adjusted odds ratios (95% CIs) of hearing loss by serum α-Klotho levels in the study population.

**Serum α-Klotho (pg/mL)**	**n**	***OR*** **(95%CI)**, ***p-*****value**	
		**SFHL**	**HFHL**
Continuous ln-transformed	1,762	0.62 (0.43, 0.89), 0.010	0.76 (0.55, 1.04), 0.085
**Quartiles**			
Q1 (< 688.8)	442	Reference	Reference
Q2 (688.8–848.8)	439	0.80 (0.58, 1.10), 0.167	0.90 (0.67, 1.21), 0.483
Q3 (649.1–1,056.3)	441	0.64 (0.45, 0.89), 0.008	0.76 (0.56, 1.02), 0.065
Q4 (>1,056.3)	440	0.64 (0.46, 0.90), 0.011	0.72 (0.53, 0.97), 0.033
*p* for trend		0.004	0.017

Multiple logistic regression models were adjusted for age, sex, race/ethnicity, BMI, physical activity, marital status, serum cotinine, hypertension, diabetes, and exposure to loud noise/music.

## 4 Discussion

Klotho, a gene associated with aging and age-related diseases, plays a significant role in various physiological processes, including the modulation of ion homeostasis and the regulation of oxidative stress (Buchanan et al., 2020; Abraham and Li, 2022). Previous studies suggested that Klotho might have protective effects on hearing health, particularly in the context of ARHL (Carpinelli et al., 2011; Yuan et al., 2018). However, the specific relationship between Klotho, hearing thresholds, and hearing loss has yet to be explored in humans. This study investigated the relationships of serum α-Klotho levels with both hearing thresholds and hearing loss among individuals aged 40–69 using NHANES data. Our findings revealed that elevated serum α-Klotho levels were found to be significantly associated with decreased hearing thresholds and a reduced risk of hearing loss. These associations remained consistent even after adjusting for potential confounding factors using multiple linear regression models. Our results suggested that this protein might have a protective effect on hearing loss. This finding is particularly pertinent given the widespread prevalence of hearing impairment among older adults and its detrimental impact on quality of life.

Our study results were consistent with a recent study (Zhou et al., 2023), which Zhou et al. (2023) claimed to analyze a sample of 1,781 individuals aged 20–69 from the NHANES 2007–2012. However, hearing tests for this age group were only conducted in the NHANES 2011–2012 cycle, and serum α-Klotho measurements were not taken for participants younger than 40 years old in the NHANES 2007–2010. This oversight suggested a lack of familiarity with the NHANES database, which might have compromised the reliability of their findings. In addition, their study purportedly covered the age range of 20–69 years (Zhou et al., 2023), whereas our study focuses on the middle-aged and elderly population aged 40–69 years. The risk of hearing loss increases significantly with age (Powell et al., 2021; Olusanya et al., 2019), making the population over 40 years more suitable for exploring the relationship between Klotho levels and hearing thresholds. Additionally, our study included a broader range of covariates and employed a RCS model to explore potential non-linear relationships. This approach offered a more nuanced analysis compared to the linear models used in the previous paper (Zhou et al., 2023), allowing us to capture more complex and detailed patterns. These methodological improvements enhanced the validity and depth of our findings.

The relationship between Klotho and hearing loss was supported by previous studies demonstrating its expression in critical structures of the inner ear, such as the stria vascularis and spiral ligament (Kamemori et al., 2002). These areas are vital for maintaining the ionic composition of endolymph, which is essential for the function of hair cells involved in auditory transduction (Patuzzi, 2011; Köppl et al., 2018). Thus, the role of Klotho in ion homeostasis suggested that its deficiency could disrupt the electrochemical gradients necessary for hearing, potentially leading to the increased hearing thresholds characteristic of ARHL. Furthermore, previous research has indicated that Klotho could regulate the activity of potassium channels (Cha et al., 2009; Huang, 2010), which are crucial for hair cell function. Additionally, lower Klotho levels were associated with increased oxidative stress and inflammation (Prud'homme and Wang, 2024), both of which could contribute to cellular damage in the cochlea. The degeneration of cochlear hair cells and the stria vascularis resulted from this oxidative damage, further exacerbating hearing loss (Someya and Prolla, 2010). However, our study was only a population-based correlation study, and the exact mechanisms required further experimental and clinical research.

The statistical analysis revealed significant trends showing that higher α-Klotho levels corresponded to lower average hearing thresholds across both speech and high-frequency ranges. These resulted corroborate previous findings suggesting a protective effect of Klotho on cochlear health (Takumida et al., 2009; Park et al., 2012; Yuan et al., 2018). Moreover, the ORs indicated that increases in serum α-Klotho were associated with reduced risks of both speech-frequency and high-frequency hearing loss. This relationship underscored the potential of Klotho as a biomarker for assessing hearing health in aging populations. What's more, the implications of our findings suggested potential therapeutic avenues for addressing hearing loss in aging populations. The modulation of Klotho expression through lifestyle changes or pharmacological interventions could serve as a promising strategy for mitigating the risks associated with ARHL. Previous studies indicated that dietary adjustments, including reducing sugar intake and alcohol consumption, may enhance Klotho levels (Ostojic et al., 2023). Since various nutritional factors have been known to impact hearing conditions, increasing the consumption of exogenous food supplements to boost Klotho levels could be a viable approach for improving hearing (Jung et al., 2019). These findings emphasized the importance of a holistic approach to health that encompasses both diet and lifestyle to promote Klotho expression and potentially improve hearing outcomes.

While the role of Klotho in aging preservation is becoming increasingly recognized, the precise mechanisms through which it exerts its protective effects remain to be fully elucidated. The involvement of Klotho in modulating inflammatory responses and oxidative stress is particularly noteworthy (Typiak and Piwkowska, 2021; Wang K. et al., 2023; Prud'homme and Wang, 2024). Elevated oxidative stress levels and inflammation are implicated in cochlear cell damage, leading to hearing impairment (Maniaci et al., 2024; Teraoka et al., 2024). By counteracting these pathological processes, Klotho might play a crucial role in preserving cochlear integrity and function. In addition, Klotho plays a role in promoting cellular repair and regeneration processes (Bian et al., 2015; Zhao et al., 2024). By enhancing the signaling pathways associated with cellular survival and function, Klotho might help preserve the health of cochlear cells in the face of age-related degeneration or damage. Collectively, these mechanisms illustrate how elevated levels of Klotho can confer protective effects against hearing loss.

Moreover, the interaction between Klotho and systemic factors influencing hearing loss cannot be overlooked. Various comorbidities such as chronic kidney disease, cardiovascular disease, and diabetes are prevalent in individuals with hearing impairment (Besser et al., 2018). The systemic implications of Klotho deficiency in these contexts merit further investigation. Understanding how Klotho interacts with these health conditions could provide insights into targeted interventions that address not only hearing loss but also its associated comorbidities.

This study possessed several notable strengths. Firstly, it utilized a representative sample of the general adult population in the US, thereby enhancing the external validity of the findings. Secondly, the researchers made adjustments for multiple potential confounders and effect modifiers, bolstering the robustness of the statistical analyses. Additionally, the α-Klotho variable was treated both as a continuous and categorical variable, broadening the scope of the investigation (Xu et al., 2020, 2021b). However, several limitations should be acknowledged. Firstly, it is important to note that the study design utilized in NHANES is cross-sectional, which inherently restricts the ability to establish causality between variables. Secondly, despite adjusting for multiple confounding factors in the statistical models, it is plausible that other influential factors, such as the potential methylation status of Klotho, may not have been fully accounted for (Zhu and Wei, 2023). Thirdly, it is crucial to consider that NHANES is a population-based survey, and sample selection may be influenced by various factors. This selection bias could potentially hinder the generalizability and accuracy of the results, thus warranting caution in their interpretation. Lastly, as surplus serum was employed to measure α-Klotho levels in NHANES, it is worth noting that repeated freeze-thaw cycles of the serum samples may introduce uncertainties that may impact the accuracy of the serum Klotho measurements (Neyra et al., 2020).

## 5 Conclusion

In conclusion, this study showed a significantly negative association between α-Klotho and hearing threshold as well as hearing loss in adults aged 40–69 years. These findings contributed to the growing body of evidence supporting the role of increased α-Klotho concentrations in mitigating hearing threshold and reducing the risk of hearing loss. While further epidemiological and experimental investigations are warranted to validate and expand upon our findings, we propose that preventive measures targeting hearing damage should focus on augmenting serum α-Klotho concentrations through interventions such as exogenous supplements.

## Data Availability

Publicly available datasets were analyzed in this study. This data can be found here: http://www.cdc.gov/nchs/nhanes/Index.htm.
